# Postpartum breast cancer: mechanisms underlying its worse prognosis, treatment implications, and fertility preservation

**DOI:** 10.1136/ijgc-2020-002072

**Published:** 2021-02-25

**Authors:** Hanne Lefrère, Liesbeth Lenaerts, Virginia F Borges, Pepper Schedin, Patrick Neven, Frédéric Amant

**Affiliations:** 1 Department of Oncology, KU Leuven University Hospitals Leuven Gasthuisberg Campus, Leuven, Flanders, Belgium; 2 Department of Gynecology, AVL NKI, Amsterdam, Noord-Holland, The Netherlands; 3 Department of Medicine, University of Colorado – Anschutz Medical Campus, Aurora, Colorado, USA; 4 Young Women’s Breast Cancer Translational Program, University of Colorado Cancer Center, Aurora, Colorado, USA; 5 Department of Cell, Developmental and Cancer Biology, Oregon Health & Science University, Portland, Oregon, USA; 6 Knight Cancer Institute, Oregon Health & Science University, Portland, Oregon, USA; 7 Department of Gynecology and Obstetrics, Katholieke Universiteit Leuven UZ Leuven, Leuven, Flanders, Belgium; 8 Multidisciplinary Breast Centre, UZ-KU Leuven Cancer Institute (LKI), Katholieke Universiteit Leuven UZ Leuven, Leuven, Flanders, Belgium; 9 Department of Gynecological Oncology, Amsterdam University Medical Centers, Amsterdam, The Netherlands

**Keywords:** carcinoma, gynecology, neoplasm metastasis

## Abstract

Breast cancers that occur in young women up to 5 to 10 years' postpartum are associated with an increased risk for metastasis and death compared with breast cancers diagnosed in young, premenopausal women during or outside pregnancy. Given the trend to delay childbearing, this frequency is expected to increase. The (immuno)biology of postpartum breast cancer is poorly understood and, hence, it is unknown why postpartum breast cancer has an enhanced risk for metastasis or how it should be effectively targeted for improved survival. The poorer prognosis of women diagnosed within 10 years of a completed pregnancy is most often contributed to the effects of mammary gland involution. We will discuss the most recent data and mechanistic insights of the most important processes associated with involution and their role in the adverse effects of a postpartum diagnosis. We will also look into the effect of lactation on breast cancer outcome after diagnosis. In addition, we will discuss the available treatment strategies that are currently being used to treat postpartum breast cancer, keeping in mind the importance of fertility preservation in this group of young women. These additional insights might offer potential therapeutic options for the improved treatment of women with this specific condition.

## Breast cancer and the effect of age and pregnancy

Breast cancer is the most frequently diagnosed cancer in women worldwide and remains the leading cause of cancer-related deaths, with the majority of deaths resulting from metastatic disease.^1^ While breast cancer rates are higher among women in more developed regions, rates are increasing in nearly every region globally.^1^ Breast cancer is a heterogenous disease that has been classified into four main molecular subtypes: luminal-A and luminal-B (expressing the estrogen receptor), and human epidermal growth factor receptor 2-enriched and basal-like.^2^ Breast cancer is increased in frequency in women during their childbearing years. Especially among postpartum women, defined here as breast cancers diagnosed up to 5 to 10 years after delivery and under 45 years of age, frequency is increased.^3^ By this definition, postpartum breast cancer is estimated to represent 50% of breast cancers arising in young mothers within 10 years of their last childbirth.^4–6^

Age at diagnosis, parity status, and breastfeeding history are among the most important risk factors for postpartum breast cancer. Older age at first birth correlates with an increased risk for postpartum breast cancer. Because of the trend to delay childbearing, incidence numbers are expected to increase. The increased incidence in young women (<45 years) matches the decrease in parity observed in this age group.^7^ A lower number of parities is another established breast cancer risk factor. In pre-menopausal breast cancers in particular, there is compelling evidence that breast cancer risk is reduced by ~7% for each full-term pregnancy and is increased by ~5% for each additional year that age at first pregnancy is delayed.^8^ Childbearing is known to have a dual effect on breast cancer risk, being associated with long-term risk reductions, following a transiently increased risk in the early postpartum period that can last up to 10 years. Risk reductions are most prominent when maternal age at first birth is below 26. When postponing first childbirth, the risk-inducing postpartum period is prolonged. The relative risk of breast cancer has been shown to decrease by 4.3% for every 12 months of breastfeeding.^8^ Risk reductions of 33% have been seen in women who have consistently breastfed for up to 2 years.^9^ The size of this decline did not seem to vary by age, menopausal status, the number of births, or age at first delivery.^8^ A lack of breastfeeding, especially among women with higher parity, has been associated with an increased risk for receptor-negative breast cancers.^10^ Although the specific cause has not yet been elucidated, studies have suggested several mechanisms by which breastfeeding might reduce breast cancer risk. Breastfeeding might prolong the process of cellular differentiation, leading to full differentiation of mammary epithelial cells.^10^ Prolonged breastfeeding is also known to decrease the amount of menstrual cycles and hence reduce hormone exposure, which could lower the risk for luminal breast cancers especially.^10^

Postpartum breast cancer that occurs 5 to 10 years' postpartum is associated with worse survival rates and an almost 2-fold increased risk for metastasis compared with breast cancers diagnosed in young, premenopausal women during and outside pregnancy.^5 6 11–13^ Several mechanisms have been proposed to explain its poor prognosis. As premenopausal women are more often diagnosed with triple-negative breast cancer compared with postmenopausal women,^6^ some attributed the poor prognosis of postpartum breast cancer to a higher incidence of this particular poor prognostic subtype. However, the frequencies of biological subtypes have not shown to differ by parity status in cohorts of young women with breast cancer.^6^ Other mechanisms that have been suggested are a delayed diagnosis due to physical changes in the breast of pregnant and lactating women, and hormonal changes during or shortly after pregnancy.^14^ Young women under the age of 45 rarely undergo screening, so their cancers are primarily self-detected, larger and more advanced than screen-detected tumors. However, these factors alone cannot fully explain the outcome differences observed between postpartum and pregnant patients. As a consequence, one or more factors unique to the immediate post-pregnancy breast microenvironment are thought to be responsible for causing the increased risk in postpartum breast cancer.^15^ Though some argue to classify postpartum breast cancer as a distinct entity, it remains unknown whether there is a molecular (and/or clinical) basis for that. This results partially from the fact that previously, postpartum breast cancer patients were often studied along with pregnant patients as ‘pregnancy-associated breast cancers’, with conflicting clinical outcome data as a result. Large epidemiologic studies and a recent meta-analysis, however, demonstrated that women specifically diagnosed and treated during pregnancy had no increased risk for metastasis and death.^3 16 17^ When we^5 6 11^ and others^12 13^ specifically studied larger cohorts of postpartum breast cancer patients diagnosed up to 10 years' postpartum separate from patients with breast cancer who were nulliparous or pregnant at diagnosis, they were found to have a significantly worse outcome. This poor prognosis persisted after the adjustment of several clinicopathological factors such as age at diagnosis, year of diagnosis, stage, grade, and hormone receptor status.^5 6^ Also, age at first birth and the number of pregnancies did not seem to further influence the prognosis of postpartum breast cancer.^6 12^ Our own analysis of almost 1200 breast cancer patient outcome data from an international cohort confirm these findings (unpublished data). Although most studies confirm this poor prognosis for postpartum cases, especially for patients diagnosed within the first year after delivery, there is also some data contradicting this.^17^ The reason for this inconsistency can be contributed to the definition of the control group as all young women with breast cancer that are diagnosed after 1 year of childbirth, while it has been shown that the poor prognosis associated with a postpartum diagnosis can last up to 10 years.^4–6^

In this review we will explore whether postpartum breast cancer, defined as breast cancer diagnosed up to 5 to 10 years in women<45 years of age, can be seen as a distinct entity with a unique biology. We will focus on the mechanisms that underly the poor prognosis of postpartum breast cancer, especially those related to mammary gland involution (regression of the mammary gland to its pre-pregnant state). In addition, we will discuss the available treatment strategies that are currently being used to treat these young women, keeping in mind the importance of fertility preservation.

## Pro- and anti-tumorigenic roles of postpartum breast involution and lactation

Due to the sparsity of human breast tissue, studies of postpartum breast cancer in women are limited and often with very small patient numbers. In the next paragraphs we will further elaborate on current knowledge with regard to the influence of involution, lactation, and the tumor microenvironment on the tumor biology of this cancer. Most evidence to date is derived from rodent models (Table 1).

**Table 1:** Studies investigating the molecular and/or immunological mechanisms during mammary gland involution in PPBC in rodent models

Rodent		
Research subject and methods	Findings	Ref
**I. Studies investigating ECM remodeling and tumor** **invasiveness**		
ECM from mammary glands of np and involuting rats. Invasive potential in metastatic MDA-MB-435 cells.	Increased motility and invasion of ECM in MDA-MB-435 cells. Increased fibronectin and MMP activity in in vitro cultured cells of the involuting mammary ECM.	Bemis^34^ 2000
ECM from mammary glands of np, pregnant, lactating, and involuting rats. Mouse mammary epithelial tumor cell line TM-6 with hormone withdrawal-induced death.	Increased fibronectin and fibronectin fragment levels in involuting ECM associated with apoptosis in TM-6. Fibronectin fragments induce ECM proteases and contribute to epithelial cells loss and dissolution of mammary alveoli in involuting ECM.	Schedin^31^ 2000
Mammary glands from np, pregnant, lactating. and involuting mice. Affymetrix microarrays.	Increased expression of components of apoptosis, inflammatory cytokines, and acute-phase response genes during involution.	Clarkson^20^ 2004
Isolated ECM from mammary glands of np, pregnant, lactating, and involuting rats. Histological, IHC, and western blotting analyzes.	Increased epithelial cell proliferation; differentiation, death, and reorganization in ECM isolated from involuting rats. Changed ECM function and fibronectin, laminin, clusterin, and MMP composition in parous mammary gland.	Schedin^28^ 2004
ECM from mammary glands of np and involuting rats. In vivo models of MDA-MB-231 cells injected into mammary fat pad.	Deceased ductal organization and increased invasiveness in tumor MDA-MB-231 cells of involuting ECM. Increased metastasis to the lung, liver, and kidney in the involution group.	McDaniel^29^ 2006
Normal mammary glands, mammary tumors, and explants in 3D culture of involuting mice. Epithelial-stromal interactions via histology, electron microscopy, and optical imaging.	Increased orientation along certain aligned collagen fibers in involuting mice, facilitating tumor invasion. Primary tumor explants realigned collagen fibers to migrate.	Provenzano^33^ 2006
In vivo rodent model of PPBC: injecting 4T1 and D2A1cells into the portal vein of involuting mice/rats. Flow cytometry and MS of mouse/rat livers.	Induced maternal liver involution characterized by hepatocyte cell death and stromal remodeling in post-weaning mice. Increased liver metastasis in post-weaning mice.	Goddard^36^ 2017
**II. Studies investigating lymphangiogenesis**		
In vivo rodent model of PPBC: injecting DCIS-GFP, D2A1, or 66cl4-LUC cells into mammary fat pad of SCID or BALC/c mice on INV1. Lymphatic vessel density by LYVE1 +vessel count and mRNA gene expression levels. COX-2 knock down cells injected into postpartum host.	Increased PPBC tumor sizes, LN positivity, lung micro-metastases, and LYVE1 +mammary lymphatic vessel density 3–4 days after weaning. Increased gene expression of Lyve1, vegfrd, vegfr2, and vegfr3 during involution. Decreased peritumor lymphatic vessel density (60%) and decreased invasion of lymphatics (85%) in shCOX-2 PPBC.	Lyons^26^ 2014
In vivo rodent model of PPBC: injecting 66cl4-DDK, E0771-DDK, 66cl4-SEMA7A, or E0771-SEMA7A-overexpressing-cells in Balb/c mice on INV1. IHC, flow cytometry, lymphangiogenesis and macrophage migration, endothelial cell adhesion, and expression of PDPN assays.	Increased expression of SEMA7A and PDPN-expressing cells in involuting tumors. Increased expression of PDPN in macrophages during involution. Increased motility and adherence of PDPN-expressing macrophages to lymphatic endothelial cells that promoted lymphangiogenesis.	Elder^38^ 2018
**III. Studies investigating the immune** **component**		
Normal mammary glands from involuting Balb/c mice that were weaned after 7 days. Affymetrix microarrays for transcript analysis of involution. Protein extracts, western blotting, and IHC.	Upregulated expression of 145 genes during first 4 days of involution, including: 49 immunoglobulin genes 12 acute-phase response genes (STAT3, CD14, LBP) Increased infiltration of neutrophils, plasma cells, macrophages, and eosinophils in involuting mammary tissue.	Stein^22^ 2004
In vivo rodent model of involution: weaned rats and C57BL/6 mice at day 10 (rats) or 14 (mice) of lactation. IHC and quantification of CD68, CSF-1R, and F4/80. ECM isolation, chemoattractant & zymogen assay, collagen detection and quantification, and collagen western blot.	Increased macrophage influx (8-fold) during involution that exhibited an M2-phenotype with expression of IL-4 and IL-13. Increased chemotactic capacity for macrophages from involuting ECM. Increased fibrillar collagen levels and proteolysis during involution. Increased chemoattractant capacity for denatured collagen I during involution.	O'Brien^45^ 2010
In vivo rodent model of PPBC: injecting D2A1 cells into mammary fat pad of mice on INV1. In vivo treatment model of PPBC: intraperitoneal injection of anti-IL-10 or rat IgG on L10, INV2, INV4, INV6, and INV8. Flow cytometry, IHC, and western blot.	Increased tumor sizes (6-fold), decreased CD4 +, and CD8+T cell infiltrates and increased number of macrophages in involuting mice. Reduced tumor growth in IL-10 targeted mice.	Martinson^44^ 2015
In vivo rodent model of involution: weaned Balb/c mice at day 9 to 13 of lactation. In vivo rodent model of PPBC: injecting FACS sorted fibroblasts and D2A1 cells in postweaning Balb/c mice. RT-qPCR, RNA sequencing, IF, and IHC analyzes.	Increased fibroblast activation during involution. Increased growth and decreased CD8 +T cell infiltration and tumor cell death in mammary tumors in the involuting-fibroblast group. Suppressed involution-fibroblast activation and tumor promotional capacity by ibuprofen treatment.	Guo^43^ 2017
In vivo rodent model of involution: weaned Balb/c-C57Bl/6 mice at day 9 to 14 of lactation. IHC, flow cytometry, Ag assays, and adoptive T-cell transfer.	Elevated mucosal CD4 +T cells within lactating and involuting glands. Increased accumulation of Th17-Treg CD4 +T cells and elevated levels of Gata3+, FoxP3+, and PD-1 +CD4+T cells in the involuting mammary gland.	Betts^46^ 2018
**IV. Studies investigating treatment options**		
In vivo rodent model of PPBC: injecting human breast tumor MCF10DCIS cells into the mammary fat pads on INV1. Treatment with celecoxib and ibuprofen.	Increased characterization of fibrillar collagen, high cyclooxygenase-2 (COX-2) expression, and invasive phenotype in PPBC tumors. Reduced promotional effects of celecoxib and ibuprofen.	Lyons^30^ 2011
In vivo rodent model of involution: weaned rats at day 10 of lactation and treated with NSAIDs on INV4, INV5, and INV6. In vivo xenograft model: injecting D2.OR cells mixed with ECM in fat pad of np mice. RT-PCR, western blot, ELISA, IHC, and imaging.	Reduced tumor growth of cells mixed with NSAID-involution ECM in PPBC mice compared with control-involution ECM in np mice. Identified tenascin-C as potential mediator of tumor progression during involution that is decreased by NSAID treatment.	O’Brien^64^ 2011
In vivo rodent model of PPBC: injecting D2A1 cells into fat pad of Balb/c-C57Bl/6 mice on INV1. In vivo multiparity mouse model of involution: weaned Balb/c-C57Bl/6 mice at day 10 of lactation (ibuprofen treated) – repeated 2 x. Flow cytometry, mass spec, immunoblot, bone marrow assays, RT-PCR, RNA seq, multiplex IHC, and TCGA.	Increased tumor growth in PPBC mice with a distinct immune milieu compared with tumor of np mice. Increased monocytes and reduced number of T-cells in PPBC mice, which is reversed on ibuprofen treatment. Enhanced Th1-associated cytokines and T-cell accumulation by ibuprofen treatment. Ibuprofen does not impede normal involution.	Pennock^65^ 2018
In vivo rodent model of involution: weaned Balb/c-C57Bl/6 mice at day 10 to 14 of lactation. In vivo rodent model of PPBC: injecting 66cl4 or E0771 carcinoma cells in Balb/c or C57Bl/6 mice on INV1. Lymphatic vessel density, multiplex staining, flow cytometry, and cytokine staining	Increased PD-1, PD-L1 expression, and PD-L1 T-cells in mouse mammary tissues during normal postpartum involution. Increased expression of CD8 +T cells expressing co-inhibitory receptors PD-1 and Lag-3 in PPBC mice models. Reduced tumor growth in postpartum mice using PD-1 targeted therapies. Reduced lymphatic vessel frequency using PD-1 therapies.	Tamburini^62^ 2019

ECM, extracellular matrix; IF, immunofluorescence; IHC, immunohistochemistry; INV1, involution day 1; LN, lymph node; MMP, matrix-metalloproteinase; MS, mass spectrometry; np, nulliparous; PDPN, podoplanin; PPBC, postpartum breast cancer.

### Involution-associated alterations in the mammary gland

During pregnancy, the mammary gland epithelium undergoes extensive proliferation and differentiation in order to prepare for lactation. After parturition in the absence of lactation, or at weaning, the mammary gland remodels to a functional and morphological state similar to pre-pregnancy, a process called involution.^18 19^ Rodent models indicate that the involution process resembles tissue-remodeling programs that are activated during wound healing. In both human and mouse mammary glands, postpartum involution begins with controlled apoptosis of mammary epithelial cells that typically occurs in two distinct phases. The first, reversible phase is characterized by the accumulation of milk globules, also known as milk stasis, and shedding of secretory mammary alveolar cells to the distended lumen.^20^ Early programmed cell death occurs 24 hours after pup removal in rodents and involution-associated gene expression changes have been noticed as early as 12 hours after weaning.^20^ Despite massive cell death that occurs during the first phase, involution can be reversed due to the expression of tissue inhibitors of metalloproteinases that are induced when lactation is resumed.^21^ In the absence of pro-survival signals, downregulation of these inhibitors results in activation of matrix metalloproteinases that degrade the extracellular matrix and activate apoptotic factors, thus comprizing the second wave of cell death.^21^ In mice, this second phase of involution has been reported to occur 48 hours after weaning and is associated with an immune cell influx, including neutrophils, macrophages, plasma cells, and eosinophils.^22^ In women, this has been confirmed by the presence of a differentially expressed immune gene-signature in the human parous breast compared with healthy nulliparous tissue that persisted up to 10 years after childbirth.^23^ The processes that occur with involution, as discussed below, are hypothesized to contribute to increased cell survival, tumor initiation, and/or growth and facilitated dissemination of pre-existing tumor cells.

During postpartum involution in both mice and humans, a minority of mammary epithelial cells survives cell death. Resistance to cell death plays an important role in tumor initiation, as it results in the propagation of potentially harmful mutations that allow unregulated growth and division.^24^ However, to this date, there is almost no available evidence that points to an increased tumor initiation caused by mammary gland involution processes. Next to potentially creating an environment that allows tumor cell initiation, the pregnancy, lactation, and involution cycle may have a long-lasting stimulatory effect on already existing tumor cells. Each cycle is characterized by immunosuppression, increased infiltration of inflammatory cytokines, and a higher tolerance to various foreign antigens.^25^ Tumour cells^26^ and mammary epithelial cells^23^ have been shown to be permanently altered after such a cycle in rodents. Hence, tumor cells that would otherwise be recognized and destroyed by the immune system have a greater chance to survive and proliferate. Although there have been more than 50 transgenic mouse models generated to date that reflect postpartum involution,^27^ the reason why some cells survive and others die remains largely unknown and further investigation is warranted.

Furthermore, available evidence, mainly supported by multiple pre-clinical animal models, points to mammary gland involution as being a driver of metastasis. Extensive apoptosis of mammary secretory epithelium, extracellular matrix remodeling, and adipocyte repopulation were shown to occur in the initial phase of mammary gland involution (Table 1 – part I).^21 28^ Extracellular matrix proteolytic fragments have been shown to participate in cell signaling events, modulate gene expression, and to directly stimulate tumor growth, motility, and invasion.^26 29 30^ The involuting microenvironment has been characterized by infiltration of macrophages, neutrophils, and plasma cells, elevated activity of matrix metalloproteinases, higher levels of extracellular matrix deposit including fibrillar collagen, and an elevation of proteolytic fragments of laminin and fibronectin.^20 22 28 31 32^ Increased extracellular matrix stiffening, collagen deposition, and cross-linking during mammary gland involution has been shown to provide a structural network for tumor cell migration, which further promotes tumor cell invasion and metastasis.^33^ In accordance with the effects of wound-healing stroma, postpartum involution was found to activate human mammary tumor cell motility and invasion in vitro.^29 34^ In addition, tumor cells in the involuting mammary gland were shown to have a metastatic preference to certain organs.^35^ Goddard et al^36^ found that the rodent liver underwent involution after weaning, as it was shown to double in size during pregnancy and lactation to allow for its increased anabolic metabolism. This process was shown to enhance metastasis to the liver during postpartum involution.^36^ Relevance in postpartum women has been obtained (Table 2 – part I), as involution was found to be characterized by wound-healing-like tissue remodeling programs that occur within 18 months after delivery.^37^ Increased deposition of collagen in postpartum breast cancer patients has also been correlated with decreased relapse-free survival.^30^ In addition, postpartum breast cancer patients were found to present with a significant increased risk in liver metastasis compared with nulliparous controls.^36^ No significant differences were found for lung, brain, and bone metastasis.

**Table 2:** Studies investigating the molecular and/or immunological mechanisms during mammary gland involution in PPBC in humans

Human		
Research subject and methods	Findings	Ref
**I. Studies investigating ECM remodeling and wound-healing-like programs**		
FISH analysis of collagen deposition and orientation in the breast tissue of 3 np and 3 PPBC women. Analysis of 11 publicly available microarray datasets of 345 cases≤45 years that relapsed.	Increased collagen deposition in PPBC. Increased COL1A1 and COX-2 expression that correlated with decreased relapse-free survival.	Lyons^30^ 2011
Analysis of adjacent normal breast tissue from 183 premenopausal women aged 20 to 45 years, grouped by reproductive categories. Stain for lobular area, lobular composition, apoptosis, and immune cell infiltration.	Increased breast epithelial area in pregnancy and lactation. Reduced mammary epithelial area in involution <12 months. Involution is characterized by wound-healing-like tissue remodeling programs that occurs within a narrow time frame (18 months).	Jindal^37^ * 2014
**II. Studies investigating lymphangiogenesis**		
Analysis of 38 postpartum and 190 np breast cancer patients≤45 years. Analysis of lymphatic vessel density in normal adjacent and breast tumor tissue measured by D2−40+vessel count.	Increased lymph node positivity in PPBC patients. No difference in HER-2 or triple-negative cases. Increased lymphatic vessel density and tumor cell invasion of lymphatics in PPBC patients.	Lyons^26^ 2014
Analysis of SEMA7A in normal breast tissue from biopsy by IHC. Kaplan–Meier analysis of SEMA7A, PDPN, and CD68 expression in >600 breast cancers, as well as ovarian, lung, and gastric cancer.	Increased expression of SEMA7A in breast tissue from women>5 years involuting. Decreased distant metastasis-free survival when SEMA7A, PDPN, and CD68 were co-expressed.	Elder^38^ 2018
**III. Studies investigating immune cell infiltration**		
Analysis of normal breast tissue from 32 postpartum (up to 10y) and 20 np women aged 18 to 45 years. LCM, RNA expression, and RT-PCR of 64 selected genes associated with involution.	Upregulated inflammation-associated genes in postpartum women. Reduced expression of ESR1, PGR, HER2, and higher expression of ESR2 in postpartum women. 14 of 64 genes from RT-PCR were differentially regulated.	Asztalos^23^ * 2010
Analysis of normal and tumor tissue from 6 np, 9 pregnant, 11 lactating, 8 involuting, and 10 regressed women aged ≤45 years. IHC, IF, imaging of CD68, CSF-1R, and F4/80.	Increased macrophage number during involution that exhibited an M2-phenotype with expression of IL-4 and IL-13.	O’Brien^45^ 2010
Analysis of tumor tissue from 17 recent pregnant, 17 distant pregnant, and 19 np breast cancer patients aged 18 to 45 years. RT-PCR, IHC, and image analysis.	Different gene expression pattern (8-gene signature) in breast cancers detected in PPBC diagnosed up to 10 years after delivery, mainly attributable to the TNBC subgroup.	Asztalos^47^ * 2015
Analysis of tumor tissue from 50 postpartum and 7 np breast cancer patients, grouped by reproductive categories. IHC of IL-10 and FoxP3.	Increased infiltration of IL-10 +and FoxP3 +immune cells in post-lactational human breast tissue suggestive of immunosuppressive microenvironment.	Martinson^44^ 2015
Analysis of tumor tissue from 3 postpartum patients and 3 np breast cancer patients. Multiplex analysis and TCGA RNAseq analysis	Increased PD-L1 expression and PD-L1 T-cells in postpartum patients. Observed co-expression of immune inhibitory PD-L1, PDPN, and CD68 in breast cancer TCGA patients.	Tamburini^62^ 2019

*Studies exclusively focusing on human breast tissue.

ECM, extracellular matrix; IF, immunofluorescence; IHC, immunohistochemistry; np, nulliparous; PPBC, postpartum breast cancer; RT-PCR, real-time PCR.

Another mechanism in the involuting gland that has been suggested to influence postpartum breast cancer prognosis is related to an increased peritumor lymphatic vessel density. Increased lymph node metastasis has been observed in postpartum involuting preclinical mouse models (Table 1 – part II).^4 26 38^ The highest vascular densities were observed at day 10 of involution in mice, followed by a slight decrease in the regressed gland. Involution-specific lymphangiogenesis in these mice was shown to allow increased infiltration of tumor cells into lymph vessels and seeding of the lymph node.^26 35 38^ This is consistent with the increased vessel density and lymph node metastasis observed in postpartum patients compared with age-matched nulliparous patients (Table 2 – part II).^6 26 38^

In support of the hypothesis that involution imprints an aggressive phenotype to postpartum breast cancer, classic weaning-induced mammary gland signatures have been reported in poor prognostic breast cancers.^39–41^ Although many of these animal models significantly contributed to an increased understanding of the molecular mechanisms involved in breast cancer development and progression in women, results should be interpreted with caution. More evidence is still needed on the effects of tumor cell imprinting and site-specific metastasis advantage in the postpartum host and their effects on breast cancer prognosis.

### Immune component of the involuting mammary gland in postpartum breast cancer

In addition to the mechanisms described before, infiltration of immune cell subsets into the mammary microenvironment is another important mechanism that has been linked to the prognosis of postpartum breast cancer. Postpartum involution is generally characterized by an initial inflammatory response followed by an immunosuppressive phase, driven by apoptosis and clearance of the secretory mammary epithelium.^15 20 22^ Immunosuppressive mechanisms might facilitate the dissemination of pre-existing tumor cells, as already being addressed before. Through identification of molecular markers for both innate and adaptive immunity within the involuting mammary gland, evidence for macrophage, neutrophil, and lymphocyte activation has been found.^20 22^ Current knowledge on the different constituents of the tumor immune microenvironment during involution in both rodents (Table 1 – part III) and women (Table 2 – part III) will be further discussed here.

Cancer-associated fibroblasts are the most common component of the tumor stroma in breast cancers and they have been found to play a critical role in the tumor microenvironment and the immune response.^42^ They are not only involved in extracellular matrix remodeling, angiogenesis, deposition of basement membrane components, and epithelial cell differentiation, but can also promote cancer initiation, progression, invasion, and metastasis.^43^ As fibroblasts are known to play an important role in wound-healing processes, and there is a strong link between these processes and cancer, they are hypothesized to play a role in postpartum breast cancer progression. Guo et al^43^ demonstrated that normal fibroblasts isolated from involuting mammary glands from mice exerted immunomodulatory functions consistent with immune suppression. They and others stated that cancer-associated fibroblasts are able to adjust immune cell infiltration and immune suppression via increased secretion of several growth factors, cytokines, and chemokines. Regarding the presence of myeloid cells during mammary gland involution, enrichments in alternatively activated macrophages and myeloid-derived suppressor cells have been noted.^44 45^ Alternatively, activated macrophages are most important in immune suppression, wound-repair mechanisms, and tissue remodeling. Through secretion of immunosuppressive cytokines such as IL-10 and TGF-β, macrophages shut down the immune system by effectively inhibiting CD8+ T cells – the dominant anti-tumor T-cell type – and dendritic cell responses. Increased presence of these immunosuppressive cytokines has already been shown in the involuting mammary gland in rodents and in breast cancer patients,^44 45^ being consistent with the promotion of alternative macrophage activation. In addition, involuting macrophages have also been shown to promote metastasis via loosening of the lymphatic vasculature.^38^ Recruitment of these macrophages is increased during mammary gland involution and accounts for the increased lymphangiogenesis in postpartum breast cancer,^4^ as described before. The lymphoid lineage during postpartum involution is typically characterized by a suppression of CD8+ T cells and an elevation of Th-17, Th-2, and regulatory CD4+ T cells in rodents.^35 44 46^ Increased accumulation of Th-17 and regulatory T-cells, and elevated levels of FoxP3+ and PD-1+ T cells, have been shown in a mice model of postpartum involution.^46^ Suppressive regulatory T-cells correlated with poor prognosis in those mice. This immune suppressive microenvironment seemed to be transient, as the immune milieu resembled the nulliparous state 6 weeks' postweaning.^46^ Low CD8+ T cell infiltration in the postpartum microenvironment resulted in tumor cell escape from immune surveillance and was also associated with decreased survival rates.^35 44^ As both immunosuppressive and immune-tolerant programs are enhanced during involution, they could be important mechanisms for mammary tumor promotion.

In women, investigations in normal post-lactational breast tissue are sparse and limited due to low patient numbers. Nevertheless, data to date show transient high IL-10 + and Foxp3+ immune cell infiltrates and the presence of T-cell suppressive macrophages, being suggestive of an immunosuppressed environment.^44 45^ Although in women the remodeling phase of mammary gland involution presumably occurs in a narrow timeframe after delivery and/or cessation of lactation, a deregulated immune profile can last up to several months or even years.^37 44^ In fact, the wound-healing-like tissue remodeling phase of involution in women has been found to occur within 18 months after delivery^37^ and distinct immune signatures have been found to persist up to 10 years after delivery.^47^ In addition, a whole genome-sequencing analysis of epithelial and stromal compartments from the normal breast has indicated that pre-existing mutated clones increase in size and undergo rapid expansion with both parity and age.^48^ These findings offer a potential explanation for the differences observed in breast cancer risk and the development of pregnant, postpartum, or nulliparous breast cancers. It is important to note that these studies lack data on lactation – thus the key window of weaning-induced involuting has yet to be defined in healthy women. Nonetheless, the persistence of deregulated immune infiltrates could possibly be explained by a positive feedback loop that is established in postpartum tumors, although evidence is lacking. In postpartum breast cancer patients, additional characterization of the tumor immune microenvironment and its influence on tumor development and progression is needed to address these remaining questions.

### Role of lactation

**Figure 1:**
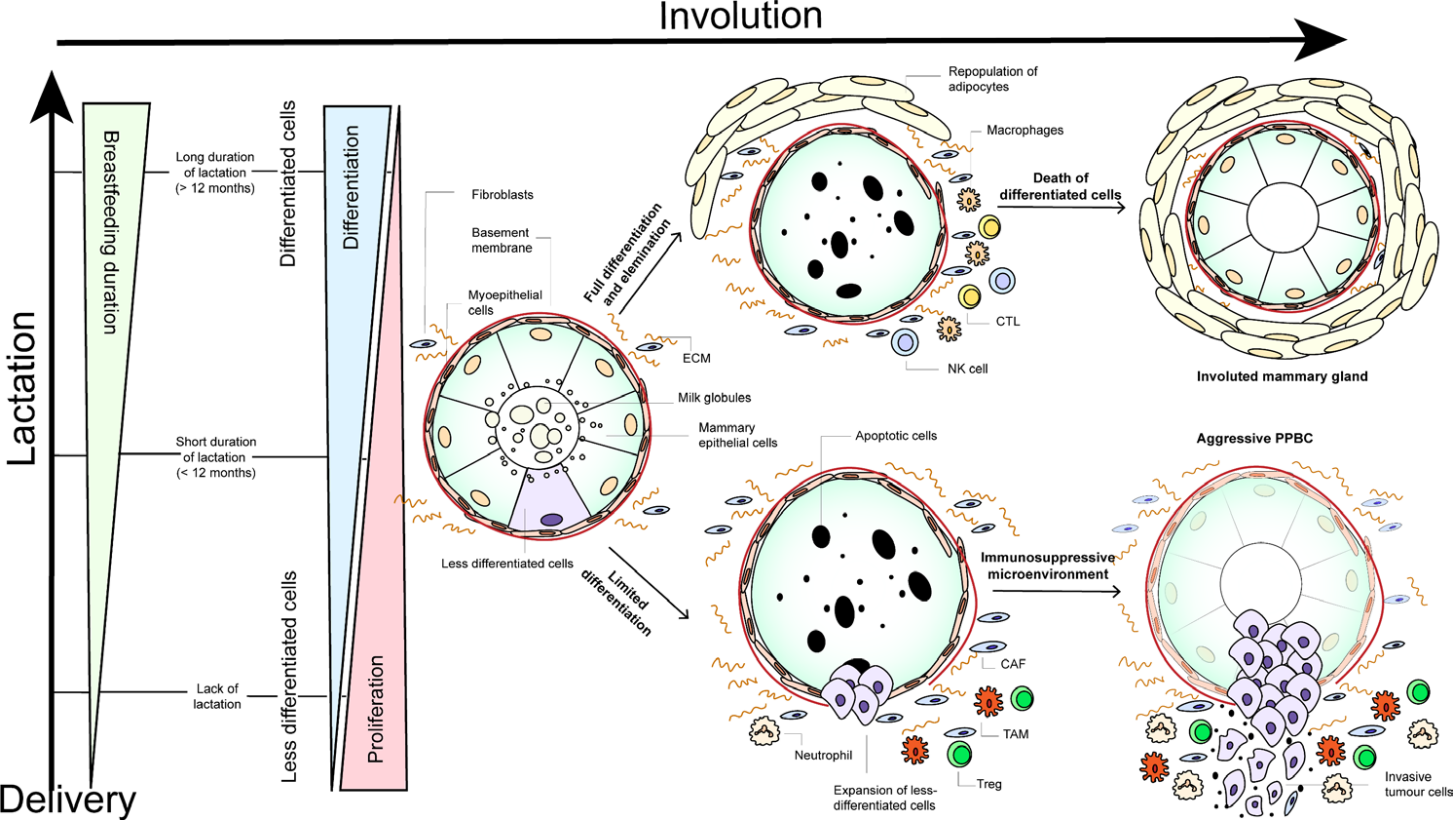
Hypothetical model depicting potential mechanisms during involution and lactation that might explain the poor prognosis of postpartum breast cancer patients. during pregnancy and lactation, Proliferation and differentiation of the mammary gland occurs. It is hypothesized that longer periods of lactation (>12 months) give rise to terminal differentiation of the majority of mammary cells.^52^During mammary gland involution, these mammary epithelial cells are subsequently removed by apoptosis, in order to return the mammary gland to its pre-pregnant state. Besides extensive apoptosis, the involution process can be characterized by infiltration of leukocytes and repopulation of adipocytes (upper panel).^21 28^ Fully differentiated cells are considered to be immune-presenting and might lead to an influx of antigen-presenting macrophages, CTLs, and NK cells.^15 52^These infiltrated immune cells might rid the mammary gland of these pro-tumorigenic cells and reduce the risk of aggressive postpartum tumor cell formation. In contrast, a lack or short duration of breastfeeding (<12 months) could give rise to less differentiated cells that are less targeted by apoptosis during the subsequent involution process.^52^Survival and expansion of these less differentiated cells might suppress the immune microenvironment associated with normal involution, which might lead to the formation of aggressive tumors with increased invasiveness (lower panel).^24^ Resistance to cell death plays an important role in tumor initiation, as it results in the propagation of potentially harmful mutations that allow unregulated growth and division.^24^ These surviving cells are thought to produce a multitude of immunosuppressive factors that might lead to the production of an immunosuppressive microenvironment comprizing neutrophils, Tregs, TAMs, and cancer-associated fibroblasts.^15^As these cells are known to modulate the immune system to an immunosuppressive state, they might become more motile and invasive, increasing the risk for metastases. increased extracellular matrix stiffening, collagen deposition, and cross-linking during mammary gland involution, mediated by the infiltration of several of these immune-related cells, has been shown to provide a structural network for tumor cell migration, which further promotes tumor cell invasion and metastasis.^33^ Extracellular matrix proteolytic fragments have also been shown to directly promote growth, motility, and invasion.^29^: PPBC, postpartum breast cancer; CTLs, cytotoxic CD8 +T cells; NK cells, natural killer cells; Tregs, regulatory T cells; TAMs, tumor-associated macrophages; CAFs, cancer-associated fibroblasts; ECM, extracellular matrix.

Whereas the protective effects of breastfeeding on the lifetime risk for breast cancer as discussed before have been well described, the association between breastfeeding and breast cancer prognosis has not been explored in many patient studies so far, and with mixed results.^3 49^ Munguía et al^49^ investigated breast cancer mortality rates in 92 794 Mexican women that were classified according to duration of breastfeeding (never,<6 months, 6 to 11 months, 12 to 23 months, and ≥24 months). They found that longer periods of breastfeeding in postpartum women could be associated with lower all-cause mortality. In contrast, Stensheim et al^3^ compared cause-specific survival between 59 pregnant, 138 non-pregnant, and 46 lactating breast cancer patients, and found a significant increased risk for cause-specific death for those that were diagnosed during the lactation period. Of note, they defined lactation as the period from the date of delivery until 6 months' postpartum to reflect the postpartum hormone changes. Whether or not these patients were actually breastfeeding at the time of diagnosis or already in involution was not investigated. Schedin and colleagues^50 51^ suggested that the effect of lactation may be related to the process of involution. Based on the evidence described above, we propose a hypothetical model that combines the role of lactation and involution mechanisms in postpartum breast cancer (Figure 1). Whether or not this model, pointing to the poor prognosis of postpartum breast cancer, might be applied to women remains unknown. Investigations in normal post-lactational breast tissue are sparse and more studies are needed to confirm these hypotheses.

From the data described here, we might conclude that postpartum breast cancer diagnosed up to 5 to 10 years after delivery is characterized with a unique biology that influences its prognosis.^52^ Extensive apoptosis of mammary secretory epithelium, extracellular matrix remodeling, immune cell infiltration, and adipocyte repopulation that occur specifically during mammary gland involution are among the most important mechanisms associated with the increased risk for metastasis and death in these patients. However, more research is warranted to identify postpartum breast cancer as a distinct entity within young women's breast cancer. Additional knowledge might also indicate whether women with postpartum breast cancer would benefit from specialized treatment strategies that specifically target this distinct biology of postpartum breast cancer.

## Current treatment options and fertility counseling in postpartum breast cancer patients

Although postpartum breast cancer is a high-risk subset of young women’s breast cancer, there are no current specific treatment guidelines. Because a breast cancer diagnosis in young women (<45 years) is often associated with high risk, healthcare providers regularly consider more aggressive treatments for these young women. However, these breast cancer therapies might result in a decreased reproductive health, ovarian-insufficiency, delayed childbearing due to prolonged treatment, breastfeeding problems, and concerns regarding future fertility.

Dependent on the biological subtype, modified radical mastectomy or systemic therapy are often seen as the first line of treatment in young patients. As no differences in outcome between mastectomy vs lumpectomy with radiation was found in more than 20 000 young women with breast cancer,^53^ modified radical mastectomy should not automatically be considered as the primary choice. Anthracyclines, cyclophosphamide, taxanes, 5-fluoroacil, and platinum agents are the most frequently administered chemotherapeutics. Because of the toxicity of chemotherapy, an important additional implication for young women is premature ovarian failure and reduced fertility.^54^ Cyclophosphamide and anthracycline or platinum treatment have been especially associated with a high to intermediate risk for infertility and premature ovarian failure. To date, not a lot of research exists on the differences in efficacy of chemotherapeutic treatments in pre- vs post-menopausal women. Subgroup analyzes have, however, indicated that the similar treatment strategies are equally efficacious at all ages,^55^ which challenges the presumption that young premenopausal women should be more often treated with chemotherapy. Gonadotropin-releasing hormone agonists are advised in young breast cancer patients as these suppress spontaneous ovulation, thereby insulating the ovary from some of the harmful effects of chemotherapy that lead to premature ovarian failure, and reduce the recurrence of luminal tumors.^54^ Hormone receptor positive patients receive endocrine therapy post-treatment for 5 years or more. Prolonged treatment has been associated with a declined reproductive capacity and tamoxifen might also harm the fetus in an unintended subsequent pregnancy. Therefore, almost 20% of young breast cancer patients stop all protocol-assigned endocrine therapy early,^56^ increasing their risk of recurrence.

As long as no other specific (targeted) therapies are identified for patients with postpartum breast cancer, treatment should, however, not differ from current guidelines advised for young women with breast cancer. As postpartum breast cancer can interrupt a woman’s life in many ways, it is important to address considerations regarding fertility and family planning with the young mother prior to initiation of treatment. Breast cancer treatment can reduce the probability of conceiving up to 70% as compared with healthy women of the same age.^57^ Postpartum breast cancer can affect women while nursing their infant and force them to undergo abrupt weaning; it can also affect women who are actively trying to conceive a child and whose plans now must be delayed; or it can affect women that have completed childbearing and who might now have to consider definitive steps to prevent further pregnancy. Informing the patient of the possible risks has been shown to relieve stress and improve quality of life.^58^ Assisted reproductive technologies, such as in vitro fertilization, can be used before the initiation of neoadjuvant chemotherapy or in the interval between surgery and chemotherapy to produce embryos, which can be stored for long periods using cryopreservation. Although currently there is no evidence that these technologies would increase the risk of recurrence, only a small percentage of patients seem to pursue available fertility preservation strategies. This might be explained by a fear of treatment delay and/or an increased risk of recurrence, as well as the cost in some countries. No increased risk for (metastatic) recurrence has, however, been related to a subsequent pregnancy in women that were previously treated for breast cancer. Pregnancy after cancer (treatment) has been associated with beneficial effects when compared with cancer survivors who did not conceive.^59^ It is not yet known why a pregnancy after cancer, rather than preceding the diagnosis, has opposite effects on breast-cancer prognosis.

## Future therapeutic options for young women with postpartum breast cancer

As postpartum breast cancer might be characterized with a distinct biology that accounts for its poor prognosis, future research could focus on novel therapeutic strategies that specifically address the biology of postpartum breast cancer (Table 1 – part IV). Given the importance of the influx of immune cells in the involuting gland, there might be a potential benefit of using immunotherapy for the preventive or therapeutic management of postpartum breast cancer. In breast cancer in general, the correlation between immune cell infiltration and poor prognosis has been well-studied, and has driven investigations into immunotherapy for breast cancer, such as vaccines and strategies targeting tumour-associated macrophages and immune checkpoint pathways such as the PD-1/PD-L1 pathway.^60^ In postpartum breast cancer patients, additional characterization of the tumor immune microenvironment and its influence on tumor development and progression is needed to address the lack of specific treatment options for this important group of young women.

A first important method to relieve immunosuppression in the tumor microenvironment is through targeting immune checkpoints. Especially receptor-negative breast cancers can be characterized with increased immune infiltration, PD-L1 expression, and tumor mutational burden and are thought to have the highest potential for immune checkpoint inhibitor-based strategies.^61^ Increased PD-L1 expression and immunosuppression has also been shown in postpartum breast cancer mouse models and might indicate the benefit of immune checkpoint inhibitors in women diagnosed within 5 to 10 years' postpartum.^62^ As involuting macrophages have been found to contribute to the poor prognosis of postpartum breast cancer, they could represent another target for immunotherapy in these patients.^63^ Reducing tumour-associated macrophage recruitment and activation has had therapeutic success in preclinical mammary cancer models where it was shown to decrease tumor growth and metastasis.^45^ An additional target in postpartum breast cancer might be macrophage chemoattractant CCL2, as its levels have been shown to be greatly increased during involution in rodent models prior to macrophage influx.^45^ Other mechanisms to target involuting macrophages are those that can tip the balance of the tumour-promotional, alternatively activated phenotype toward classical activated macrophages with increased anti-tumor attributes. Candidate cytokines for ‘re-education’ of these macrophages include IL-4, IL-13, IL-10, and TGF-β. Another way to reduce immunosuppression in the tumor microenvironment of the involuting mammary gland is through targeting with general anti-inflammatory agents. Nonsteroidal anti-inflammatory drug treatment in animal models of postpartum breast cancer has been identified as a safe potential strategy for prevention and treatment through macrophage differentiation, T-cell recruitment, and tumor suppression.^30 64 65^ Additional trials are warranted to investigate the beneficial effects of nonsteroidal anti-inflammatory drugs, possibly in combination with immunomodulatory therapy, for women with postpartum breast cancer.

Further investigations of the involuting breast microenvironment may define pathways implicated in postpartum breast cancer tumorigenesis and metastasis, facilitating the development of new therapeutic agents for prevention and treatment.

## Conclusion

Postpartum breast cancer diagnosed in young women (<45 years) up to 5 to 10 years after delivery has been associated with an increased risk for metastasis and death. This poor prognosis has been correlated to the distinct biology of postpartum breast cancer, which arises from processes related to lactation (duration) and mammary gland involution. Despite accumulating evidence from preclinical animal models into the factors underlying the aggressive biology of postpartum breast cancer, the exact mechanisms that lead to the poor prognosis of postpartum breast cancer in humans remains largely unknown. Additional insight is needed to improve prognosis and quality of life in this important group of young women. A first potential mechanism underlying its aggressive biology might be the immunosuppressive state that has been observed during postpartum involution. Patterns of altered immune infiltration have been found to persist in the primary tumor microenvironment for several years after delivery.^15 20 22^ Another mechanism that remains largely unexplored is how and when existing indolent tumor cells disseminate from the involuting microenvironment. It might require several years for the indolent tumor and the disseminated tumor cells to become clinically detectable. Most evidence up to now arises from animal models. To draw a more general conclusion for postpartum breast cancer, it has to be elucidated how information from these animal models can be translated to the human setting. There is evidence that especially the immune system differs between mice and humans,^66^ and results should thus be interpreted with caution. Weaning in animal models is generally a rapid process where pups are abruptly removed from the mother, whereas in women it is usually a more gradual process. The length of lactation and/or the timing of weaning (prolonged vs rapid) may influence the duration of involution, and, therefore, affect gene expression programs and components of the microenvironment. In mice, an expansion of murine mammary epithelial cells has been seen during pregnancy with a consequent a drop below baseline levels after weaning.^67^ Similar mechanisms might take place in the human breast, however, the scarcity of human breast tissue during pregnancy, lactation, and involution poses considerable challenges for conducting such studies in humans. In addition, the interaction between reproductive factors and breast cancer is complex, making it hard to differentiate between mechanisms related to pregnancy, uniparity, multiparity, age at first birth, lactation, involution, and the breast tumor itself. Expanded efforts to collect reproductive data and to explore the biology of breast cancer during pregnancy, lactation, and involution in both healthy and tumor tissue of young women are urgently required. As breastfeeding seems to be inversely related to both breast cancer risk and prognosis, an additional focus on lactation programs is needed. Both retrospective and prospective analyzes of breastmilk could potentially identify additional biomarkers shed into the milk that might be associated with increased recurrence or mortality.

Future research should especially focus on finding evidence of a postpartum signature in women with a postpartum breast cancer diagnosis, which could be based on gene expression data in human tissue and/or blood. Improved understanding of the pathways underlying the increased metastatic rate and the identification of molecular and/or cellular biomarkers with prognostic and/or predictive value in humans is a prerequisite for exploring optimized therapeutic modalities for postpartum breast cancer.

## References

[1] WHO-International. Breast cancer, 2018.

[2] Perou CM , Sørlie T , Eisen MB , et al. Molecular portraits of human breast tumours. Nature 2000;406:747–52. 10.1038/35021093 10963602

[3] Stensheim H , Møller B , van Dijk T , et al. Cause-specific survival for women diagnosed with cancer during pregnancy or lactation: a registry-based cohort study. J Clin Oncol 2009;27:45–51. 10.1200/JCO.2008.17.4110 19029418

[4] Borges VF , Elder AM , Lyons TR . Deciphering pro-lymphangiogenic programs during mammary involution and postpartum breast cancer. Front Oncol 2016;6:1. 10.3389/fonc.2016.00227 27853703 PMC5090124

[5] Callihan EB , Gao D , Jindal S , et al. Postpartum diagnosis demonstrates a high risk for metastasis and merits an expanded definition of pregnancy-associated breast cancer. Breast Cancer Res Treat 2013;138:549–59. 10.1007/s10549-013-2437-x 23430224 PMC3608871

[6] Goddard ET , Bassale S , Schedin T , et al. Association between postpartum breast cancer diagnosis and metastasis and the clinical features underlying risk. JAMA Netw Open 2019;2:e186997. 10.1001/jamanetworkopen.2018.6997 30646210 PMC6484560

[7] Lima SM , Kehm RD , Swett K , et al. Trends in parity and breast cancer incidence in US women younger than 40 years from 1935 to 2015. JAMA Netw Open 2020;3:e200929. 10.1001/jamanetworkopen.2020.0929 32167569 PMC7070232

[8] Collaborative Group on Hormonal Factors in Breast Cancer. Breast cancer and breastfeeding: collaborative reanalysis of individual data from 47 epidemiological studies in 30 countries, including 50302 women with breast cancer and 96973 women without the disease. Lancet 2002;360:187–95. 10.1016/S0140-6736(02)09454-0 12133652

[9] Li CI , Beaber EF , Tang M-TC , et al. Reproductive factors and risk of estrogen receptor positive, triple-negative, and HER2-neu overexpressing breast cancer among women 20-44 years of age. Breast Cancer Res Treat 2013;137:579–87. 10.1007/s10549-012-2365-1 23224237 PMC3547981

[10] do Carmo França-Botelho A , Ferreira MC , França JL , et al. Breastfeeding and its relationship with reduction of breast cancer: a review. Asian Pac J Cancer Prev 2012;13:5327–32. 10.7314/APJCP.2012.13.11.5327 23317179

[11] Van den Rul N , Han SN , Van Calsteren K , et al. Postpartum breast cancer behaves differently. Facts Views Vis Obgyn 2011;3:183–8. 24753864 PMC3991453

[12] Whiteman MK , Hillis SD , Curtis KM , et al. Reproductive history and mortality after breast cancer diagnosis. Obstet Gynecol 2004;104:146–54. 10.1097/01.AOG.0000128173.01611.ff 15229014

[13] Strasser-Weippl K , Ramchandani R , Fan L , et al. Pregnancy-associated breast cancer in women from Shanghai: risk and prognosis. Breast Cancer Res Treat 2015;149:255–61. 10.1007/s10549-014-3219-9 25504083 PMC6613825

[14] Al-Amri AM . Clinical presentation and causes of the delayed diagnosis of breast cancer in patients with pregnancy associated breast cancer. J Family Community Med 2015;22:96–100. 10.4103/2230-8229.155383 25983605 PMC4415134

[15] Schedin P . Pregnancy-associated breast cancer and metastasis. Nat Rev Cancer 2006;6:281–91. 10.1038/nrc1839 16557280

[16] Hartman EK , Eslick GD . The prognosis of women diagnosed with breast cancer before, during and after pregnancy: a meta-analysis. Breast Cancer Res Treat 2016;160:347–60. 10.1007/s10549-016-3989-3 27683280

[17] O'Sullivan CC , Irshad S , Wang Z , et al. Clinico-pathologic features, treatment and outcomes of breast cancer during pregnancy or the post-partum period. Breast Cancer Res Treat 2020;180:695–706. 10.1007/s10549-020-05585-7 32162192 PMC7398490

[18] Werb Z , Sympson CJ , Alexander CM , et al. Extracellular matrix remodeling and the regulation of epithelial-stromal interactions during differentiation and involution. Kidney Int Suppl 1996;54:S68–74. 8731199 PMC2937007

[19] Watson CJ , Kreuzaler PA . Remodeling mechanisms of the mammary gland during involution. Int J Dev Biol 2011;55:757–62. 10.1387/ijdb.113414cw 22161832

[20] Clarkson RWE , Wayland MT , Lee J , et al. Gene expression profiling of mammary gland development reveals putative roles for death receptors and immune mediators in post-lactational regression. Breast Cancer Res 2004;6:R92–109. 10.1186/bcr754 14979921 PMC400653

[21] Watson CJ . Involution: apoptosis and tissue remodelling that convert the mammary gland from milk factory to a quiescent organ. Breast Cancer Res 2006;8:203 10.1186/bcr1401 16677411 PMC1557708

[22] Stein T , Morris JS , Davies CR , et al. Involution of the mouse mammary gland is associated with an immune cascade and an acute-phase response, involving LBP, CD14 and STAT3. Breast Cancer Res 2004;6:R75–91. 10.1186/bcr753 14979920 PMC400652

[23] Asztalos S , Gann PH , Hayes MK , et al. Gene expression patterns in the human breast after pregnancy. Cancer Prev Res 2010;3:301–11. 10.1158/1940-6207.CAPR-09-0069 20179293

[24] Hanahan D , Weinberg RA . Hallmarks of cancer: the next generation. Cell 2011;144:646–74. 10.1016/j.cell.2011.02.013 21376230

[25] Froehlich K , Schmidt A , Heger JI , et al. Breast cancer, placenta and pregnancy. Eur J Cancer 2019;115:68–78. 10.1016/j.ejca.2019.03.021 31121525

[26] Lyons TR , Borges VF , Betts CB , et al. Cyclooxygenase-2-dependent lymphangiogenesis promotes nodal metastasis of postpartum breast cancer. J Clin Invest 2014;124:3901–12. 10.1172/JCI73777 25133426 PMC4153700

[27] Radisky DC , Hartmann LC . Mammary involution and breast cancer risk: transgenic models and clinical studies. J Mammary Gland Biol Neoplasia 2009;14:181–91. 10.1007/s10911-009-9123-y 19404726 PMC2693781

[28] Schedin P , Mitrenga T , McDaniel S , et al. Mammary ECM composition and function are altered by reproductive state. Mol Carcinog 2004;41:207–20. 10.1002/mc.20058 15468292

[29] McDaniel SM , Rumer KK , Biroc SL , et al. Remodeling of the mammary microenvironment after lactation promotes breast tumor cell metastasis. Am J Pathol 2006;168:608–20. 10.2353/ajpath.2006.050677 16436674 PMC1606507

[30] Lyons TR , O'Brien J , Borges VF , et al. Postpartum mammary gland involution drives progression of ductal carcinoma in situ through collagen and COX-2. Nat Med 2011;17:1109–15. 10.1038/nm.2416 21822285 PMC3888478

[31] Schedin P , Strange R , Mitrenga T , et al. Fibronectin fragments induce MMP activity in mouse mammary epithelial cells: evidence for a role in mammary tissue remodeling. J Cell Sci 2000;113 (Pt 5:795–806. 10671369 10.1242/jcs.113.5.795

[32] Goddard ET , Hill RC , Barrett A , et al. Quantitative extracellular matrix proteomics to study mammary and liver tissue microenvironments. Int J Biochem Cell Biol 2016;81:223–32. 10.1016/j.biocel.2016.10.014 27771439 PMC5459605

[33] Provenzano PP , Eliceiri KW , Campbell JM . Collagen reorganization at the tumor-stromal interface facilitates local invasion. BMC Med 2006;4:1. 10.1186/1741-7015-4-38 17190588 PMC1781458

[34] Bemis LT , Schedin P . Reproductive state of rat mammary gland stroma modulates human breast cancer cell migration and invasion. Cancer Res 2000;60:3414–8. 10910049

[35] Borges VF , Lyons TR , Germain D , et al. Postpartum involution and cancer: an opportunity for targeted breast cancer prevention and treatments? Cancer Res 2020;80:1790–8. 10.1158/0008-5472.CAN-19-3448 32075799 PMC8285071

[36] Goddard ET , Hill RC , Nemkov T , et al. The rodent liver undergoes weaning-induced involution and supports breast cancer metastasis. Cancer Discov 2017;7:177–87. 10.1158/2159-8290.CD-16-0822 27974414 PMC5459606

[37] Jindal S , Gao D , Bell P . Postpartum breast involution reveals regression of secretory lobules mediated by tissue-remodeling. Breast Cancer Res 2014;16:1. 10.1186/bcr3633 24678808 PMC4053254

[38] Elder AM , Tamburini BAJ , Crump LS . Semaphorin 7A promotes macrophage-mediated lymphatic remodeling during postpartum mammary gland involution and in breast cancer. Cancer Res 2018;78:6473–6485. 10.1158/0008-5472.CAN-18-1642 30254150 PMC6239927

[39] Stein T , Salomonis N , Nuyten DSA , et al. A mouse mammary gland involution mRNA signature identifies biological pathways potentially associated with breast cancer metastasis. J Mammary Gland Biol Neoplasia 2009;14:99–116. 10.1007/s10911-009-9120-1 19408105

[40] Bambhroliya A , Van Wyhe RD , Kumar S , et al. Gene set analysis of post-lactational mammary gland involution gene signatures in inflammatory and triple-negative breast cancer. PLoS One 2018;13:e0192689. 10.1371/journal.pone.0192689 29617367 PMC5884491

[41] Santucci-Pereira J , Zeleniuch-Jacquotte A , Afanasyeva Y , et al. Genomic signature of parity in the breast of premenopausal women. Breast Cancer Res 2019;21:46:1–19. 10.1186/s13058-019-1128-x 30922380 PMC6438043

[42] Liu T , Han C , Wang S , et al. Cancer-associated fibroblasts: an emerging target of anti-cancer immunotherapy. J Hematol Oncol 2019;12:86:1–15. 10.1186/s13045-019-0770-1 31462327 PMC6714445

[43] Guo Q , Minnier J , Burchard J , et al. Physiologically activated mammary fibroblasts promote postpartum mammary cancer. JCI Insight 2017;2:e89206. 10.1172/jci.insight.89206 28352652 PMC5358521

[44] Martinson HA , Jindal S , Durand-Rougely C , et al. Wound healing-like immune program facilitates postpartum mammary gland involution and tumor progression. Int J Cancer 2015;136:1803–13. 10.1002/ijc.29181 25187059 PMC4324053

[45] O'Brien J , Lyons T , Monks J , et al. Alternatively activated macrophages and collagen remodeling characterize the postpartum involuting mammary gland across species. Am J Pathol 2010;176:1241–55. 10.2353/ajpath.2010.090735 20110414 PMC2832146

[46] Betts CB , Pennock ND , Caruso BP , et al. Mucosal immunity in the female murine mammary gland. J Immunol 2018;201:734–46. 10.4049/jimmunol.1800023 29884705 PMC6036228

[47] Asztalos S , Pham TN , Gann PH , et al. High incidence of triple negative breast cancers following pregnancy and an associated gene expression signature. Springerplus 2015;4:710:1–9. 10.1186/s40064-015-1512-7 26618099 PMC4653130

[48] Cereser B , Tabassum N , Del Bel Belluz L , et al. Mutational landscapes of normal breast during age and pregnancy determine cancer risk. bioRxiv 2020. 10.1101/2020.09.04.277715

[49] Munguía MU , Esparza SL , Stern D . Breastfeeding duration and the risk of all-cause and breast cancer mortality among parous women from the Mexican teachers’ cohort. J Glob Oncol 2018;4:1–27. 10.1200/jgo.18.21000

[50] Basree MM , Shinde N , Koivisto C , et al. Abrupt involution induces inflammation, estrogenic signaling, and hyperplasia linking lack of breastfeeding with increased risk of breast cancer. Breast Cancer Res 2019;21:80:1–18. 10.1186/s13058-019-1163-7 31315645 PMC6637535

[51] Takabatake Y , Oxvig C , Nagi C , et al. Lactation opposes pappalysin-1-driven pregnancy-associated breast cancer. EMBO Mol Med 2016;8:388–406. 10.15252/emmm.201606273 26951623 PMC4818749

[52] ElShamy WM . The protective effect of longer duration of breastfeeding against pregnancy-associated triple negative breast cancer. Oncotarget 2016;7:53941–50. 10.18632/oncotarget.9690 27248476 PMC5288234

[53] Smith BD , Bellon JR , Blitzblau R , et al. Radiation therapy for the whole breast: Executive summary of an American Society for Radiation Oncology (ASTRO) evidence-based guideline. Pract Radiat Oncol 2018;8:145–52. 10.1016/j.prro.2018.01.012 29545124

[54] Abdel-Razeq H . Gonadotropin-releasing hormone agonists during chemotherapy for ovarian function and fertility preservation for patients with early-stage breast cancer. Cancer Manag Res 2019;11:4273–82. 10.2147/CMAR.S204069 31190993 PMC6514123

[55] Azim HA , Davidson NE , Ruddy KJ . Challenges in treating premenopausal women with endocrine-sensitive breast cancer. Am Soc Clin Oncol Educ Book 2016;35:23–32. 10.1200/EDBK_159069 27249683

[56] Saha P , Regan MM , Pagani O , et al. Treatment efficacy, adherence, and quality of life among women younger than 35 years in the International Breast Cancer Study Group text and soft adjuvant endocrine therapy trials. JCO 2017;35:3113–22. 10.1200/JCO.2016.72.0946 PMC5597253 28654365

[57] Peccatori FA , Azim HA , Orecchia R , et al. Cancer, pregnancy and fertility: ESMO clinical practice guidelines for diagnosis, treatment and follow-up. Ann Oncol 2013;24 Suppl 6:6:vi160–70. 10.1093/annonc/mdt199 23813932

[58] Borges VF . Management of the patient with postpartum breast cancer. Oncology 2014;28:768–70. 25224474

[59] Azim HA , Kroman N , Paesmans M , et al. Prognostic impact of pregnancy after breast cancer according to estrogen receptor status: a multicenter retrospective study. J Clin Oncol 2013;31:73–9. 10.1200/JCO.2012.44.2285 23169515 PMC3530692

[60] Choi J , Gyamfi J , Jang H , et al. The role of tumor-associated macrophage in breast cancer biology. Histol Histopathol 2018;33:133–45. 10.14670/HH-11-916 28681373

[61] Gaynor N , Crown J , Collins DM . Immune checkpoint inhibitors: key trials and an emerging role in breast cancer. Semin Cancer Biol 2020. 10.1016/j.semcancer.2020.06.016. [Epub ahead of print: 02 Jul 2020]. 32623044

[62] Tamburini BAJ , Elder AM , Finlon JM , et al. PD-1 blockade during post-partum involution reactivates the anti-tumor response and reduces lymphatic vessel density. Front Immunol 2019;10:1313:1–16. 10.3389/fimmu.2019.01313 31244852 PMC6579890

[63] O'Brien J , Schedin P . Macrophages in breast cancer: do involution macrophages account for the poor prognosis of pregnancy-associated breast cancer? J Mammary Gland Biol Neoplasia 2009;14:145–57. 10.1007/s10911-009-9118-8 19350209 PMC2693782

[64] O'Brien J , Hansen K , Barkan D , et al. Non-steroidal anti-inflammatory drugs target the pro-tumorigenic extracellular matrix of the postpartum mammary gland. Int J Dev Biol 2011;55:745–55. 10.1387/ijdb.113379jo 22161831

[65] Pennock ND , Martinson HA , Guo Q . Ibuprofen supports macrophage differentiation, T cell recruitment, and tumor suppression in a model of postpartum breast cancer. J Immunother Cancer 2018;6:98:1–23. 10.1186/s40425-018-0406-y 30285905 PMC6167844

[66] Mestas J , Hughes CCW . Of mice and not men: differences between mouse and human immunology. J Immunol 2004;172:2731–8. 10.4049/jimmunol.172.5.2731 14978070

[67] Tiede B , Kang Y . From milk to malignancy: the role of mammary stem cells in development, pregnancy and breast cancer. Cell Res 2011;21:245–57. 10.1038/cr.2011.11 21243011 PMC3193434

